# 2799!!

**Published:** 1870-02

**Authors:** 


### 2799!!

Multiplied by five, as the average number of a family, make thirteen thousand nine hundred and ninety-five hearts left crushed and bleeding, dispirited and sad, by old eighteen hundred and sixty-nine, as the business wrecks of the year. It means that two thousand seven hundred and ninety-nine men began the year either with fortunes made or in hopeful expectancy. One of them we have known for long years; a score of years ago he was a rich man, rich enough to retire from business, and he was then but forty. Twenty-five years earlier he came to New York a penniless boy. We asked him one day how it was that he so soon amassed a fortune, every dollar of which he had made himself in legitimate business, while there were multitudes in the city who had toiled even longer, had grown gray in their struggles, and there was urgent necessity for struggling still, with no very bright prospects of its ending this side the grave. "Oh," said he, "I always saw for myself that a thing was done." He had many men under him, to whom daily instructions had to be given; but he never left his business place at night until he had satisfied himself that every thing had been properly attended to; and what he could not supervise himself, he would not undertake. He had no implicit confidence in any man but himself.

As an illustration, he went on to say: "A few weeks ago I was applied to, to become the president of an insurance company about to be organized."

"I can not undertake it, for my time is already fully employed."

"The business will be attended to by others, and you will have nothing to do but to sign your name now and then to the reports of the treasurer, and some others of minor importance."

"But, then, I would make myself responsible to the community for the correctness of his accounts, which I could not consistently do without first examining every item, and ascertaining for myself that there were proper vouchers for every dollar."

"The directors will do that; and they have authorized me to offer you five thousand dollars a year, as they want the influence of your name, with little or no work for you to do."

"My dear sir, I would not act as president for any institution at a salary of fifty thousand dollars a year, and risk my business name, unless I knew for myself, at the end of every day, that all was right."

Not long after that, a ship had taken fire and went to the bottom, containing an immense sum of gold, consigned to him as his own property. We met a business man a few days later, and inquired if our friend had lost all that money.

"Lost! He never met with a loss in his life, for he never risked any thing; every dollar was insured, and the companies have already arranged to make it up."

To-day he is poorer than he was forty-five years ago, for then he was young, hopeful, ambitious, and strong in his own determination, owing not a dollar; to-day he is old, without a penny, owing immense sums. Within a week, not knowing what had happened, we met his devoted and loving wife. Two things surprised us. For twenty years we had met her at home and on the avenue, and there was always a quick and cordial greeting. But now the face was half averted, and there was such a faint smile, only a sardonic semblance, and with a word or two she passed hurriedly on; and then, again, it was in an out-of-the-way street; but we passed on, and other things crowded the incident out of mind, until next morning the whole thing came up vividly to the memory, on reading at the breakfast table: "It becomes our painful task to announce the failure of the time-honored house of —— ——." To be princely rich for a quarter of a century; to have lived in the most magnificent avenue in the world, with liveried footmen and splendid equipages; to change all for a one-story house in a by-street; to pass an old age in poverty; to walk in narrow, noisome lanes instead of driving in the Park; and more than all, and worse than all, to suffer the neglects of those who were friends in sunshine, trusted friends! Ah, there is the bitterness, to be endured for many weeks, for years, for the remainder of a life. But multiplying this suffering and sadness by the number at the head of this article, and then these two thousand seven hundred and ninety-nine wives had their sorrow and mortification doubled by as many husbands; and then all their sons and daughters; and to these may be added, in many cases, aged fathers and widowed mothers, to weep tears of blood for their sons' failures; it is perfectly terrible to think of. No wonder that merchants' wives and merchants' daughters crowd our lunatic asylums, and merchants' sons seek a suicide's grave, or, worse than that, abandon themselves to vice, and bestiality, and crime

Need we tell the reader how our friend failed; a man of such industry, holding such maxims in business, who never drank a drop, and whose private life was without a spot? It is but the old story; in the excitements of Wall Street he went outside of his legitimate business. The man who in twenty-five years of business life never ran a risk, did, in the parlance of the street, "fly a kite," and risk once, only once in twenty-five years, and lost all!

Young man, "stick to the last." Stick to your business; the business you were brought up to. If, in looking around you, you see others seemingly getting on faster than you are, don't blame your business; it is not the fault of your business, if it is legitimate and useful; the fault is in yourself; put more energy into it. Let the people know that you are living; let the people know what you are doing, where you do it, and that you do it well. We really do not know of any business, useful in itself, that will not make a man rich in a large city, or even a small one, if he follows it well and keeps at it; for every day makes him more a master of his calling; and remember, if you can't succeed in a calling which you know all about, how is it possible for you to succeed in one which you know nothing about?

